# A nomogram for predicting prognosis for young cervical neuroendocrine carcinoma: A SEER-based study and external validation

**DOI:** 10.3389/fonc.2025.1463422

**Published:** 2025-01-31

**Authors:** Ning Xie, Haijuan Yu, Jie Lin, Sufang Deng, Linying Liu, Yang Sun

**Affiliations:** Department of Gynecology, Clinical Oncology School of Fujian Medical University, Fujian Cancer Hospital, Fuzhou, Fujian, China

**Keywords:** neuroendocrine carcinoma of the cervix, youth, SEER database, prognostic nomogram, external validation

## Abstract

**Background:**

Neuroendocrine carcinoma of the cervix (NECC) is a rare and highly aggressive subtype of cervical carcinomas with poor prognosis. NECC tends to occur in young age which could severely impair mental and physical health of young patients. Therefore, this study aims to develop an individualized prognostic nomogram for young NECC patients.

**Methods:**

360 young (≤45 years old) NECC patients were retrospectively selected from the Surveillance, Epidemiology and End Results (SEER) database and were randomly located to a training cohort and an internal validation cohort in a ratio of 7:3. Data from Fujian Cancer Hospital was used as an external validation cohort. Independent prognostic factors were identified by univariate and multivariate Cox regression analysis, and a prognostic nomogram for young NECC was developed. The predictive accuracy and clinical utility of the nomogram were assessed by area under the time-dependent receiver operating characteristic (timeROC) curve (AUC), the concordance index (C-index), calibration plots, and decision curve analysis (DCA). Finally, a simplified scoring system for clinical use was constructed by dividing patients into high-risk and low-risk groups.

**Results:**

Pathological type, FIGO stage, and surgery were independent risk factors by univariate and multivariate analysis (*P* < 0.05). The prognostic nomogram consisting of the above three independent risk factors had high accuracy. The AUC values of 5-year overall survival (OS) in the training, internal validation, and external validation cohorts were 0.805, 0.798 and 0.872, respectively. The prognostic nomogram also presented with good C-index and calibration plots. The DCA curve further confirmed that the nomogram had a high clinical net benefit. According to the median prognostic index (median PI=18.6), all patients were categorized into high-risk group and low-risk group. The 5-year OS of the high-risk NECC group was significantly worse than that of the low-risk group among three cohorts (*P*<0.05).

**Conclusions:**

Pathological type, FIGO stage, and surgery were identified as independent prognostic risk factors for young NECC patients. Based on the nomogram, gynecologic oncologists can accurately and easily predict the prognosis of young NECC and provide scientific guidance for individualized treatment.

## Introduction

Neuroendocrine carcinoma of the cervix (NECC) is a rare and highly aggressive subtype of cervical carcinomas, accounting for 0.9%-1.5% of cervical cancer (CC) cases (1). It is characterized by aggressive behavior and poor prognosis, particularly in the case of mixed types, and is strongly associated with HPV infection (2). Due to its rarity and the lack of large randomized controlled trials, the treatment of NECC is mainly based on experience of cervical adenocarcinoma and squamous cell carcinoma and small cell neuroendocrine carcinoma (SCNEC) of the lung (3). However, NECC patients have a higher risk of death than patients with other pathological types of cervical cancer like cervical squamous cell carcinoma and cervical adenocarcinoma (4). A meta-analysis of 3538 of NECC showed that the 5-year overall survival (OS) was 34% (5), compared with a 5-year OS of 62.34% for all CC (6). Consequently, individualized prognostic risk assessment of NECC patients is crucial, as it can help clinicians guide appropriate treatment interventions.

Patients with NECC tend to be diagnosed at a young age (4). Vale et al. found a higher incidence rate was seen in NECC patients 25 years or younger compared with older patients (PR:6.10, 95%CI: 2.03-18.35) (7). Another retrospective study from Japan showed that the median age at diagnosis of patients with NECC was earlier than that of squamous cervical cancer and adenocarcinoma of cervix (43 vs 55 vs 51 years) (8). Given the longer life expectancy and greater tolerance to cancer treatments in young patients (9), a prognostic nomogram for young NECC patients is valuable to better tailor therapies.

However, few studies have investigated the individualized prognostic risk factors of young NECC patients up to now. Therefore, this study aims to develop the first individualized prognostic model with an external validation to help clinicians improve the prognosis of young NECC patients.

## Materials and methods

### Patient selection

**Figure 1:**
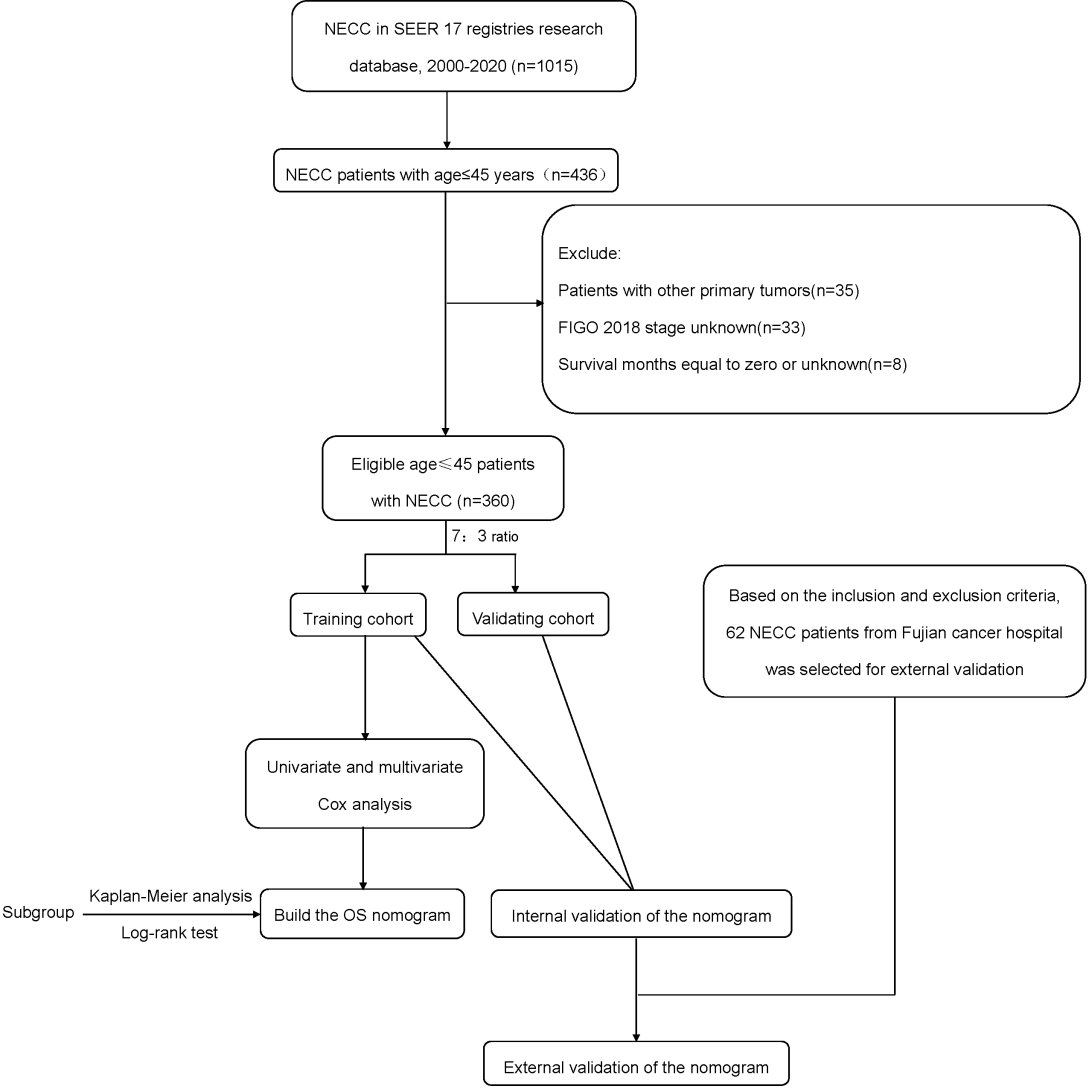
The flow chart of the study.

This study utilized a retrospective research methodology. Young (≤45 years) patients diagnosed with NECC between 2000 and 2020 were retrieved from the Surveillance, Epidemiology and End Results (SEER)-18 database using SEER*Stat software (version 8.4.3). Inclusion criteria (1): International Classification of Diseases for Oncology, 3rd edition (ICD-O-3) histology codes of 8013/3, 8041/3, 8042/3, 8045/3, 8240/3, 8241/3, 8244/3, 8245/3, and 8246/3 (2). Primary sites were C53.0, C53.1, C53.8, and C53.9 (3). Diagnosis confirmed by pathology (4). Patients aged 18-45 years at diagnosis (5). Complete clinical data and follow-up information. Exclusion criteria (1): Patients with other primary tumors (2). FIGO 2018 stage unknown (3). Survival months equal to zero or unknown. Based on the above inclusion and exclusion criteria, the external validation cohort included young NECC patients at Fujian Provincial Cancer Hospital between January 2012 and December 2023. **Figure 1** shows the flow chart of the study. Experiments involving humans were carried out following the ethics policy approved by the Ethics Committee of Fujian Cancer Hospital (Approval No. K2024-121-01).

### Data collection

Clinicopathological information collected from patients included race, marital status, age at diagnosis, pathological type, grade, tumor size, FIGO 2018, surgery, radiotherapy, and chemotherapy. The primary endpoint of was OS, defined as the time from pathological diagnosis to death from any cause or the last follow-up. All follow-up information was acquired by telephone, inpatient records and outpatient records.

### Statistics analysis

All analyses were analyzed by SPSS 26.0 and R software (version 4.3.0, http://www.r-project.org). Patients in the SEER database were randomized into a training cohort and an internal validation cohort according to a 7:3 ratio. The optimal cut-off value for age was determined based on the time-dependent receiver operating curve (timeROC). The χ2 test or Fisher exact test was used to analyze the differences in baseline characteristics between the groups. Univariate and multivariate Cox regression analysis were employed to estimate prognostic risk factors of young NECC. The predictive accuracy of the prognostic nomogram was determined by the area under the ROC curve (AUC) and consistency index (C-index). Calibration ability was assessed by the calibration plots. Decision curve analysis (DCA) was used to assess the clinical utility. According to the nomogram, the prognostic index (PI) of each patient was calculated. The patients were divided into low-risk and high-risk groups based on the PI score. Survival differences between the two subgroups were assessed by Kapla-Meier curves and Log-rank tests. Results were presented as hazard ratios (HRs) with 95% confidence intervals (CIs). All statistical tests were two-sided, and *P* values less than 0.05 were considered statistically significant.

## Results

### Clinical characteristics of patients

360 young (≤45 years old) NECC patients in the SEER database were randomly divided into the training cohort (n=251) and the internal validation cohort (n=109). External validation was performed using 62 young NECC patients from Fujian Cancer Hospital. **Table 1** summaries the clinicopathology characteristics of the training, internal validation and external validation cohorts. The median follow-up was 103 months in the training cohort, 100 months in the internal validation cohort and 67 months in the external validation cohort. The median age in each cohort was 37 (range: 21-45 years), 37 (range: 22-45 years) and 39 (range: 25-45 years), respectively.

**Table 1:** Clinicopathology of young NECC patients.

	Training cohort (n = 251)	Internal validation cohort (n = 109)	External validation cohort (n = 62)
Age (years)			
≤38	151 (60.16%)	65 (59.63%)	28 (45.16%)
>38	100 (39.84%)	44 (40.37%)	34 (54.84%)
Race			
White	190 (75.70%)	78 (71.56%)	0 (0.00%)
Black	24 (9.56%)	13 (11.93%)	0 (0.00%)
Others	37 (14.74%)	18 (16.51%)	62 (100.00%)
Marital status			
Single	93 (37.05%)	45 (41.28%)	0 (0.00%)
Married	122 (48.61%)	49 (44.95%)	62 (100.00%)
Others	36 (14.34%)	15 (13.76%)	0 (0.00%)
Pathological type			
LCNEC	27 (10.76%)	9(8.26%)	2 (3.23%)
SCNEC	152 (60.56%)	72 (66.06%)	30 (48.39%)
Others	72(28.69%)	28 (25.69%)	30 (48.39%)
Tumor size			
≤4cm	93 (37.05%)	29 (26.61%)	31 (50.00%)
>4cm	103 (41.04%)	52 (47.71%)	25 (40.32%)
Unknown	55 (21.91%)	28 (25.69%)	6 (9.68%)
FIGO 2018			
I	93 (37.05%)	41 (37.61%)	16 (25.81%)
II	13 (5.18%)	4 (3.67%)	21 (33.87%)
III	73 (29.08%)	28 (25.69%)	19 (30.65%)
IV	72 (28.69%)	36 (33.03%)	6 (9.68%)
Grade			
I/II/III	119 (47.41%)	52 (47.71%)	36 (58.06%)
IV	46 (18.33%)	22 (20.18%)	2 (3.23%)
Unknown	86 (34.26%)	35 (32.11%)	24 (38.71%)
Surgery			
No/Unknown	107 (42.63%)	46 (42.20%)	16 (25.81%)
Yes	144 (57.37%)	63 (57.80%)	46 (74.19%)
Radiotherapy			
No/Unknown	90 (35.86%)	44 (40.37%)	15 (24.19%)
Yes	161 (64.14%)	65 (59.63%)	47 (75.81%)
Chemotherapy			
No/Unknown	34 (13.55%)	15 (13.76%)	0 (0.00%)
Yes	217 (86.45%)	94 (86.24%)	62 (100.00%)

### The univariate and multivariate Cox analysis

The univariate analysis showed that age, pathological type, tumor size, FIGO 2018, and surgery were significantly associated with the prognosis of young NECC (*P*<0.05). However, only pathological type, FIGO 2018, and surgery were independent risk factors by multivariate analysis (*P*<0.05) (**Table 2**). For young NECC patients, SCNEC and surgery were independent prognostic protective factors, while FIGO stage was prognostic risk factors.

**Table 2:** Univariate and multivariate Cox regression analysis of OS in young NECC patients.

	Univariate analysis		Multivariate analysis	
	Hazard ratio (95% CI)	*P* value	Hazard ratio (95% CI)	*P* value
Age (years)				
≤38	Reference		Reference	
>38	1.534 (1.106 - 2.128)	0.010	1.261 (0.899 - 1.769)	0.179
Race				
White	Reference			
Black	1.605 (0.973 - 2.648)	0.064		
Others	0.955 (0.593 - 1.539)	0.850		
Pathological type				
LCNEC	Reference		Reference	
SCNEC	0.509 (0.311 - 0.834)	0.007	0.404 (0.241 - 0.675)	< 0.001
Others	0.697 (0.413 - 1.175)	0.014	0.542 (0.316 - 0.929)	0.026
Marital status				
Single	Reference			
Married	0.769 (0.539 - 1.096)	0.147		
Others	0.930 (0.573 - 1.510)	0.770		
Tumor size				
≤4cm	Reference		Reference	
>4cm	1.699 (1.167 - 2.474)	0.006	1.032 (0.670 - 1.592)	0.885
Unknown	1.697 (1.078 - 2.672)	0.022	1.124 (0.687 - 1.837)	0.642
Grade				
I/II/III	Reference			
IV	0.851 (0.540 - 1.341)	0.487		
Unknown	0.940 (0.653 - 1.352)	0.737		

### Development and validation of nomogram

**Figure 2:**
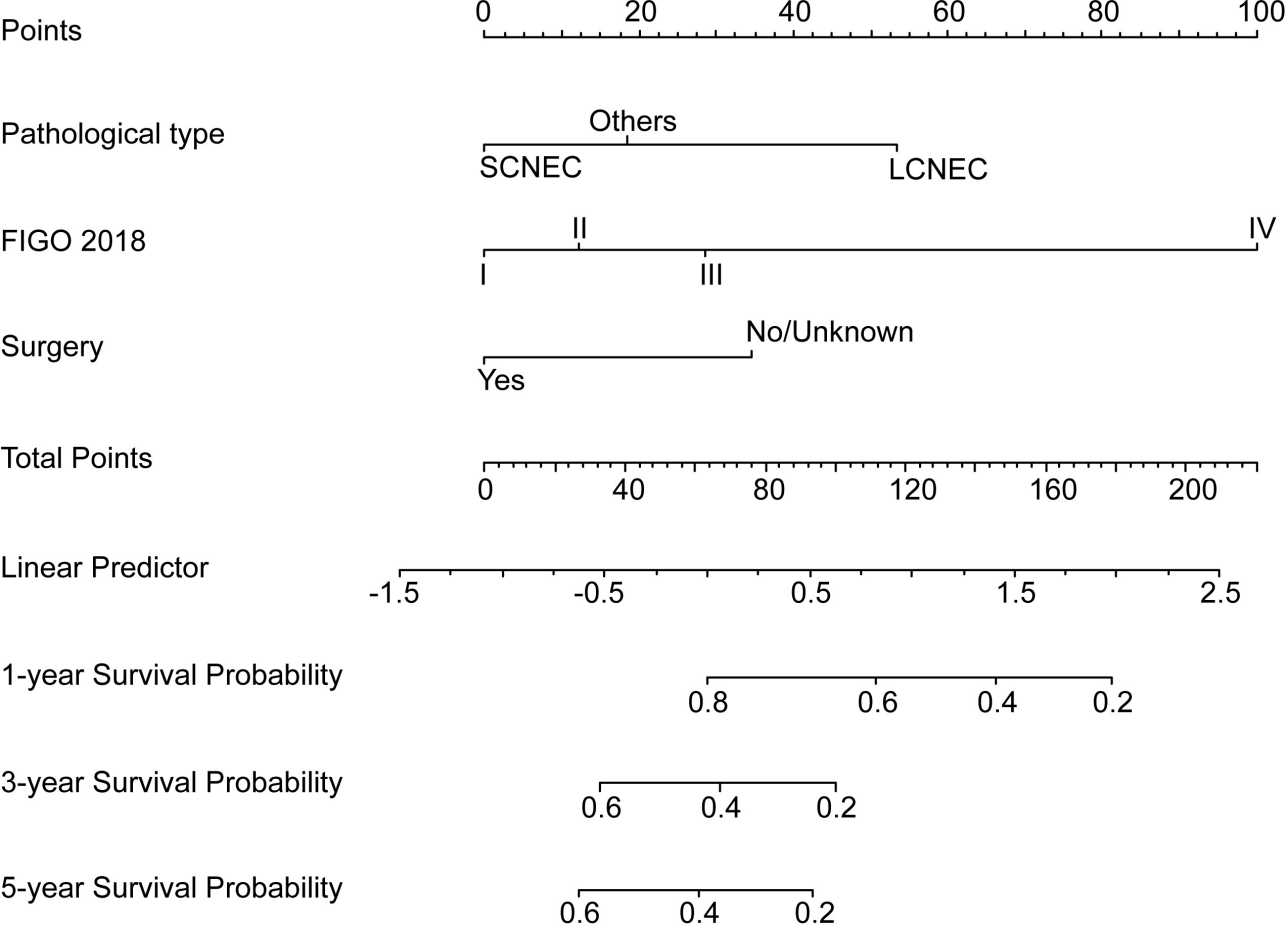
Prognostic nomogram for young NECC.

**Figure 3:**
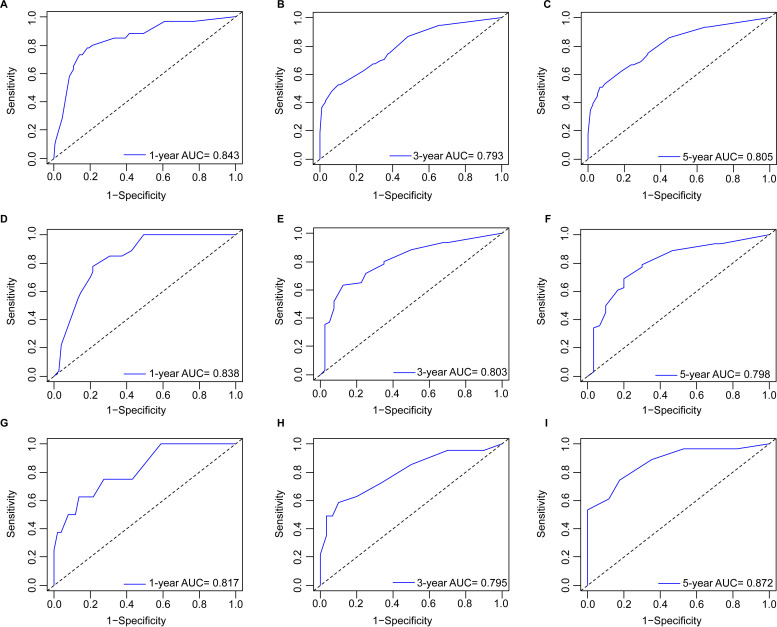
ROC curves by nomogram for 1-, 3-, and 5-year OS in young patients with NECC: **(A–C)** the training cohort; **(D–F)** the internal validation cohort; **(G–I)** the external validation cohort.

**Figure 4:**
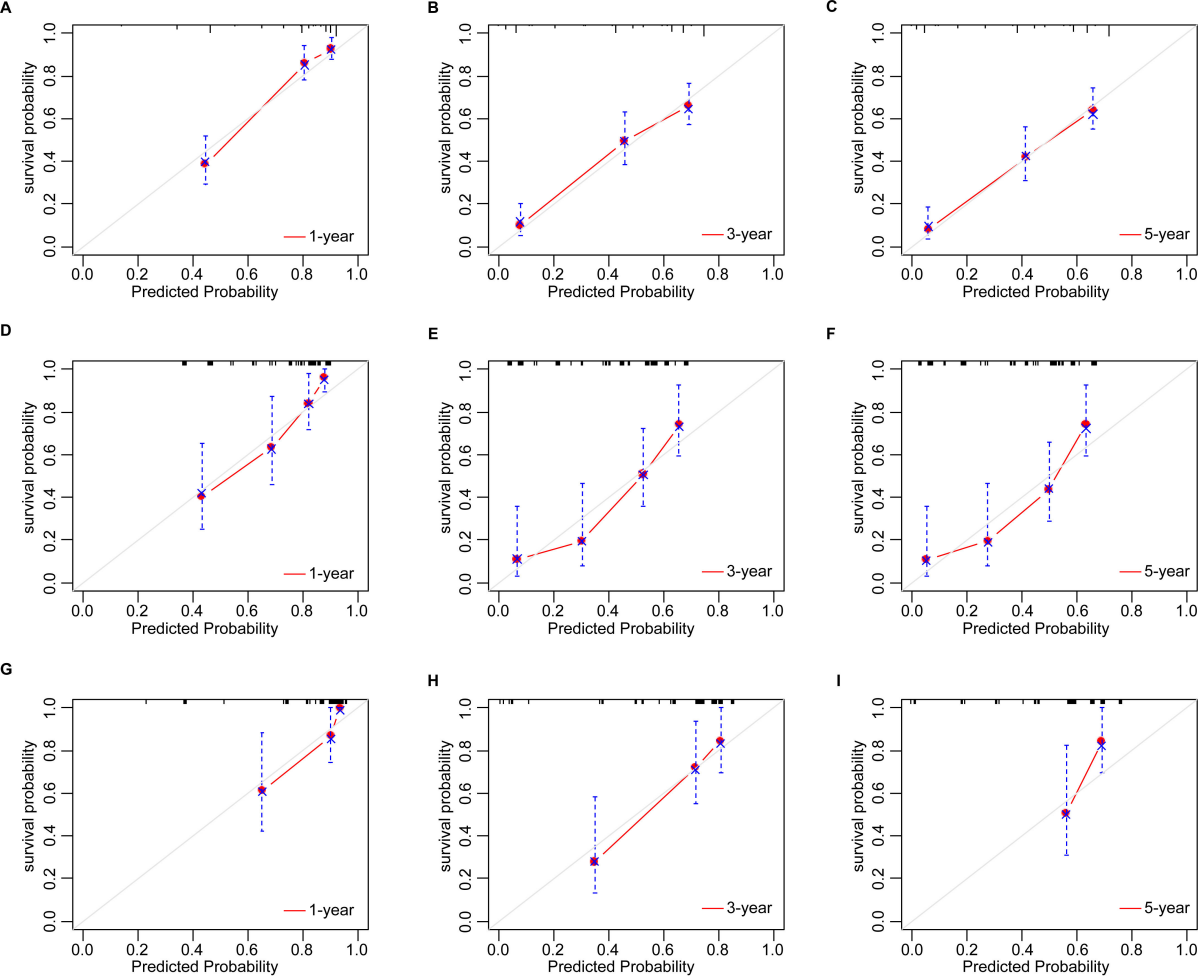
The calibration plots by nomogram for 1-, 3-, and 5-year OS in young patients with NECC: **(A–C)** the training cohort; **(D–F)** the internal validation cohort; **(G–I)** the external validation cohort.

**Figure 5:**
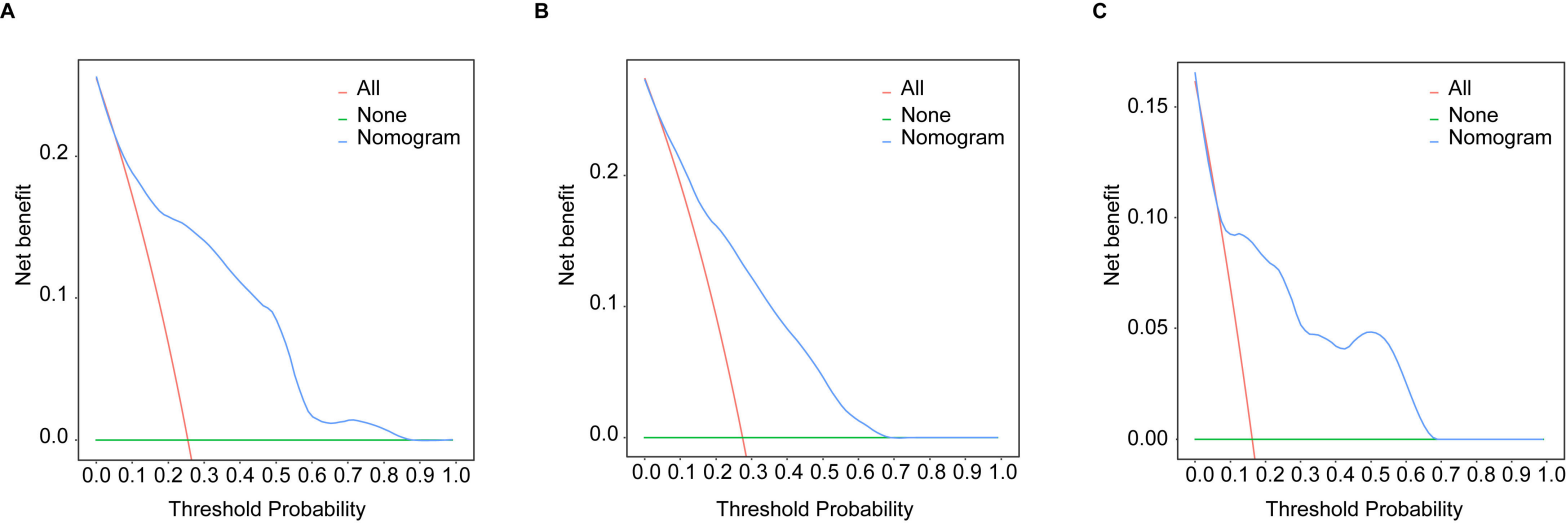
The DCA curves by nomogram in young patients with NECC: **(A)** the training cohort; **(B)** the internal validation cohort; **(C)** the external validation cohort.

Based on the multivariate Cox analyses, FIGO 2018, pathological type, and surgery were included in the development of the prognostic nomogram (**Figure 2**). The nomogram has a relatively high predictive accuracy. The AUC of 1-, 3-, and 5-year OS of the training cohort were 0.843 (95%CI: 0.783-0.903), 0.793 (95%CI: 0.737-0.849), and 0.805 (95%CI: 0.747-0.862), respectively (**Figures 3A–C**). In the internal validation cohort, the AUC for predicting 1-, 3-, and 5-year OS were 0.838 (95%CI: 0.762-0.915), 0.803 (95%CI: 0.717-0.890), and 0.798 (95%CI:0.700-0.896) (**Figures 3D–F**). Similarly, the AUC for 1-, 3-, and 5-year OS of the external validation cohort were 0.817 (95%CI: 0.665- 0.970), 0.795 (95%CI: 0.668-0.922) and 0.872 (95%CI: 0.772-0.972) (**Figures 3G–I**). The C-index of nomogram was 0.745 (95%CI: 0.706-0.784), 0.744 (95%CI: 0.689-0.784), 0.755 (95%CI: 0.665-0.845) in the training, internal validation and external validation cohorts, respectively. The calibration curves showed good agreement between the predicted and the actual probability (**Figure 4**). In addition, the DCA plots demonstrated favorable positive net benefit of the nomogram (**Figure 5**).

### Simplification of the scoring system

**Figure 6:**
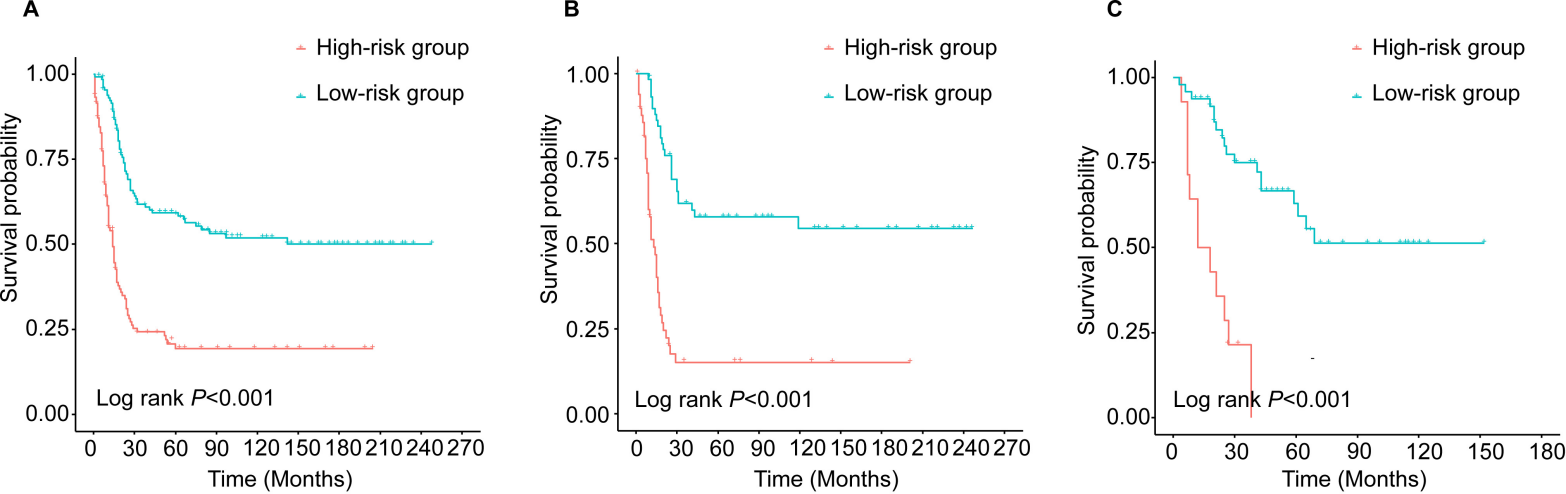
Kaplan-Meier survival curves of young patients with NECC in different risk groups: **(A)** training cohort; **(B)** internal validation and **(C)** external validation.

For clinical use, a simplified scoring system was created based on the nomogram. The PI of each patient was the sum of those risk factors score, ranging from 0 to 187.9. According to the median PI (median PI=18.6), all young NECC patients were categorized into high-risk group and low-risk groups, respectively. (**Figure 6**). The result showed that the prognosis of high-risk group was significantly worse than that of low-risk group (*P*<0.05). The 5-year OS rates of the low-risk and high-risk groups were 59.2% and 19.2% in the training cohort. In the internal validation cohort, the 5-year OS rates were 57.8% and 15.0% in the low-risk and high-risk groups, respectively. For low-risk and high-risk groups in the external validation cohort, the 5-year OS rates were 63.0% and 0.0%.

## Discussion

The results of this study showed that pathological type, FIGO stage, and surgery were independent prognostic factors for young NECC patients. Based on the above three factors, an individualized nomogram was developed to predict the prognosis of young NECC patients. The prognostic nomogram demonstrated the superior predictive ability in the internal and external validation. The calibration plots and the DCA curves further confirmed that the nomogram had a high clinical net benefit.

Our study showed that surgery was an independent prognostic risk factor in young NECC patients (HR=0.564, *P*=0.004), which is consistent with previous studies (10). Lin et al (11) found that early-stage cervical SCNEC who underwent radical surgery had a better overall survival than those who received radical radiotherapy (*P* = 0.03), and simultaneous integrated boost (SIB) was used in patients who could not be undergo surgery (12). Additionally, several studies have reported that surgery is beneficial for the prognosis of patients with advanced NECC. A multicenter retrospective study involving 678 patients with cervical SCNEC showed that surgery was not only associated with a better prognosis for patients in IB-IIA2 stages (HR 0.17, 95%CI: 0.05-0.50), but was also a protective factor for locally advanced NECC (HR 0.59, 95% CI: 0.37 - 0.95) (13). For NECC patients with oligometastases, in addition to stereotactic body radiation therapy (SBRT) therapy (14), surgery also helps to prolong survival outcomes by reducing tumor load and metastasis (15). It has been generally recommended that NECC patients receive radical hysterectomy with adjuvant chemoradiotherapy regardless of pathological risk factors (16). Therefore, the question has been raised whether radical hysterectomy can be replaced by less aggressive surgical approaches in the multimodal treatment of NECC patients to minimize postoperative morbidity. Zeng et al (17) concluded that total hysterectomy or radical hysterectomy was not associated with survival prognosis in the multimodal treatment of all-stage NECC. A study of 64 young NECC patients found that ovarian conservation had no effect on disease-free survival (DFS) and OS (18). What’s more, long-term survival has been reported in patients with stage IB1 NECC who underwent radical abdominal trachelectomy in combination with adjuvant chemotherapy (19, 20). Exploring the feasibility of less aggressive surgical approaches is significant and should be investigated in future studies to reduce postoperative morbidity or preserve the organ in young NECC patients.

Pathological type is an important independent risk factor for the prognosis of young NECC (21). According to the 5th edition of the World Health Organization (WHO) classification, neuroendocrine tumors of the female reproductive system are divided into well-differentiated neuroendocrine tumors (NETs) and poorly differentiated neuroendocrine carcinomas (NECs) (22). Among NECs, SCNEC is the most common, followed by LCNEC (22). Previous studies have shown that the 5-year OS of NECC is significantly worse than that of squamous cell carcinoma and adenocarcinoma (23). However, given the rarity of NECC, few studies have explored the prognostic differences between different pathologic types of NECs, and the results of the studies are still controversial. SCNEC is generally considered to be more aggressive than LCNEC and has a worse prognosis. A study of high-grade neuroendocrine carcinomas of the gastrointestinal carcinoma showed that the 5-year OS of LCNEC was significantly higher than that of SCNEC (32% vs 6%, *P* < 0.05) (24). However, our data showed that cervical LCNEC was associated with a worse patient prognosis than cervical SCNEC. A supportive study also found that median survival for endometrial LCNEC was much lower than for endometrial SCNEC (8 months vs 25 months) (25). Moreover, Shao et al (26) found that ovarian LCNEC had a worse 5-year OS compared to SCNEC (21.8% vs 28.0%). It suggests that in gynecological tumors, LCNEC may be associated with a worse prognosis for patients and the mechanism needs to be further investigated.

Our research showed that FIGO stage was the most important independent prognostic risk factor for young NECC. Advanced clinical stage is often considered an independent prognostic risk factor for CC (27), which is consistent with our study. It has been reported that median OS for patients with early stage (IA1-IB2) cervical SCNEC was 31.2 months, compared with 6.4 months for patients with advanced stage (IIB-IV) (28). Cohen et al. found that the 5-year disease-free survival (DSS) of patients with FIGO I-IIA, FIGO IIB-IVA, and FIGO IVB stage cervical SCNEC was 36.8%, 9.8%, and 0%, respectively (*P* < 0.001) (29). Additionally, a retrospective study based on the National Cancer Database included 1,896 NECC patients and found that 5-year OS decreased from 55% in patients with stage IB to 24% in patients with stage IIIB (4). The possible reasons may be related to the higher tumor burden in advanced NECC compared to early-stage patients. And patients with advanced NECC tend to receive palliative care regimens, which have longer treatment cycles and are more disruptive to normal bodily functions.

A new simplified scoring system was constructed based on these three independent prognostic factors, which could discriminate between high- and low-risk NECC patients. For high-risk young NECC patients, traditional treatments frequently fail to improve their prognosis, prompting consideration of innovative therapeutic options like targeted therapy and immunotherapy to improve clinical outcomes (30, 31). Paraghamian et al (32) documented a case of an individual with recurrent, metastatic, and programmed death-ligand 1 (PD-L1) negative metastatic cervical SCNEC who achieved a complete response to nivolumab. Another patient with chemotherapy refractory stage IV cervical LCNEC who was treated with a PD-L1 inhibitor in combination with stereotactic body radiotherapy (SBRT), exhibited nearly complete disease resolution (33). Carroll et al (3) found that most high-grade NECC tumor tested expressed poly ADP-ribose polymerase (PARP)-1, suggesting that PARP inhibitors might be an effective treatment. Overall, immunotherapy and targeted therapy are promising treatments for high-risk young NECC patients to improve their prognosis, and further prospective studies are needed to confirm the efficacy of these therapies.

This is the first large retrospective cohort study to investigate prognostic risk factors in young NECC patients. Using the innovative prognostic nomogram, gynecological oncologists can determine the survival of young NECC patients. However, our study has several limitations. First, as a retrospective study, selection bias was inevitable. Second, the SEER database did not contain information on chemotherapy regimens and cycles, radiotherapy dose, targeted therapy, and immunotherapy, which may also affect the prognosis of young NECC. Future, multicenter retrospective and prospective studies will be conducted to validate our findings.

## Conclusion

This study clarified that pathological type, FIGO stage, and surgery were independent risk factors for the prognosis of young NECC patients. The novel prognostic nomogram and risk stratification is expected to become an individualized and accurate tool for gynecological oncologists to evaluate the survival of young NECC patients. For high-risk young NECC patients, novel and effective therapeutic options such as targeted therapy and immunotherapy are being considered to improve the clinical prognosis.

## Data Availability

The raw data supporting the conclusions of this article will be made available by the authors, without undue reservation.
